# Supplementary material for: Regulation of Notch output dynamics via specific E(spl)-HLH factors during bristle patterning in Drosophila
Source: Nat Commun. 2019 Aug 2;10:3486. doi: 10.1038/s41467-019-11477-2 (PMC6677740; doi:10.1038/s41467-019-11477-2)
Supplement: Supplementary file 1 — Supplementary Information [file 41467_2019_11477_MOESM1_ESM.pdf]

Regulation of Notch output dynamics by specific E(spl)-HLH factors during bristle patterning in *Drosophila*

Couturier *et al.*

Supplementary Figures

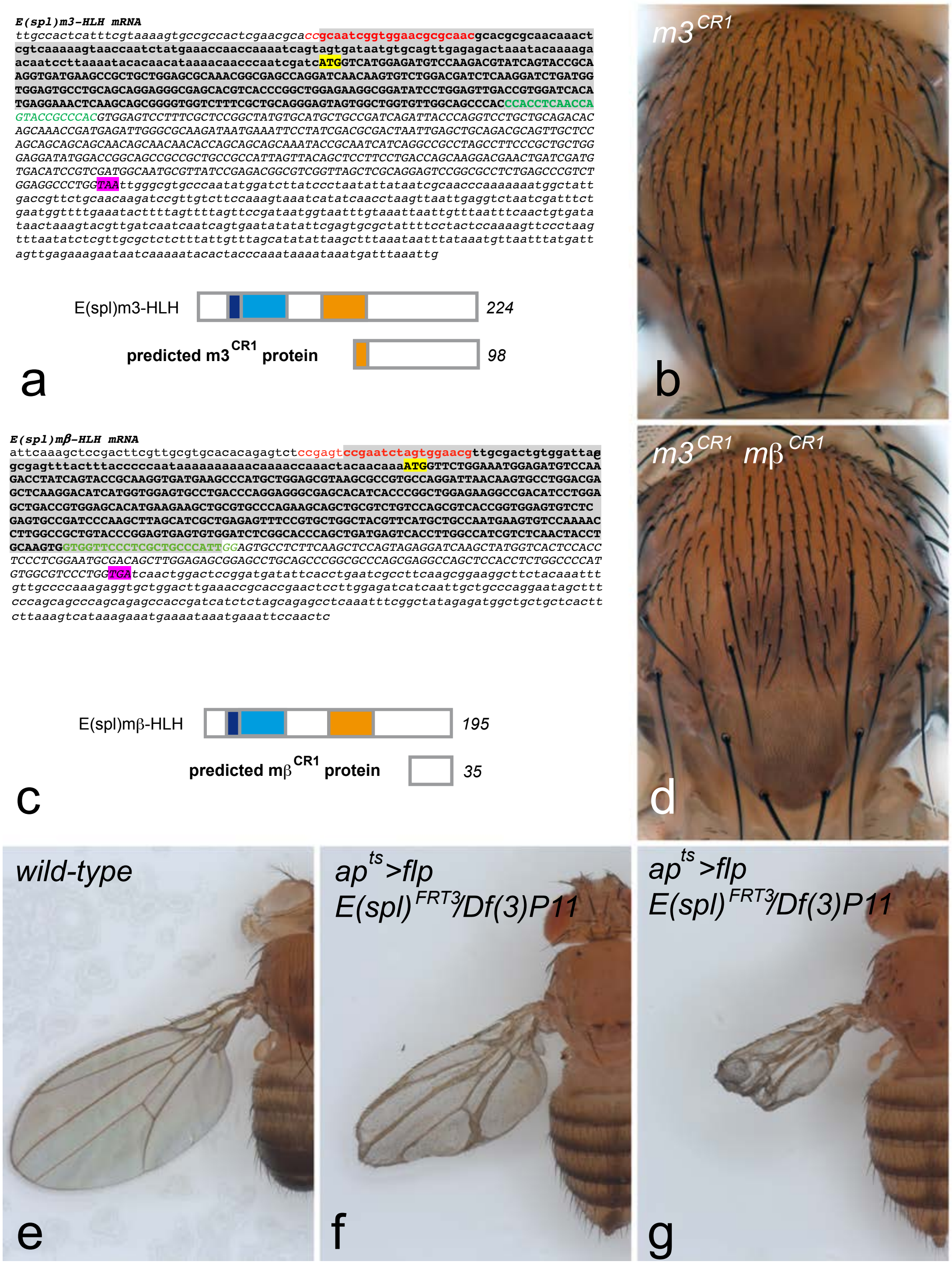

Supplementary Figure 1: *E(spl)-C* phenotypes in adult flies

a-d) Structure of the wild-type and mutant *m3* (a) and *mβ* (c) alleles with corresponding gene products, and associated notum phenotype (b, *m3*^{CR1}; d, *m3*^{CR1} *mβ*^{CR1}). The positions

of the two gRNAs (red and green) are shown on the sequence of the wild-type mRNAs (a,c). The ORFs are in capital letters (ATG, yellow; stop codon, magenta). The CRIPSR-induced deletions are shown in grey. The structure of the predicted mutant proteins (ORF in the correct phase) is shown underneath (basic, dark blue; HLH, blue; Orange domain, orange). These mutations appear to be molecularly null (a,c). The nota of $m3^{CR1}$ (b) and $m3^{CR1}$ $m\beta^{CR1}$ (d) mutant flies showed no bristle patterning defects. Loss of the *m3* alone or in combination with $m\beta$ resulted in viable flies with no detectable phenotype.
e-g) micrographs showing a wild-type fly (e) and two *Df(3)P11/* $E(spl)\text{-}C^{FRT3}$ ap^{ts}*>flp* flies showing small but otherwise properly patterned wings (f,g). Wing veins were significantly enlarged. Note that conditional deletion led to a small wing phenotype of variable expressivity (f,g).
Scale bar, 10μm. Representative images of >6 samples from >2 experiments are shown.

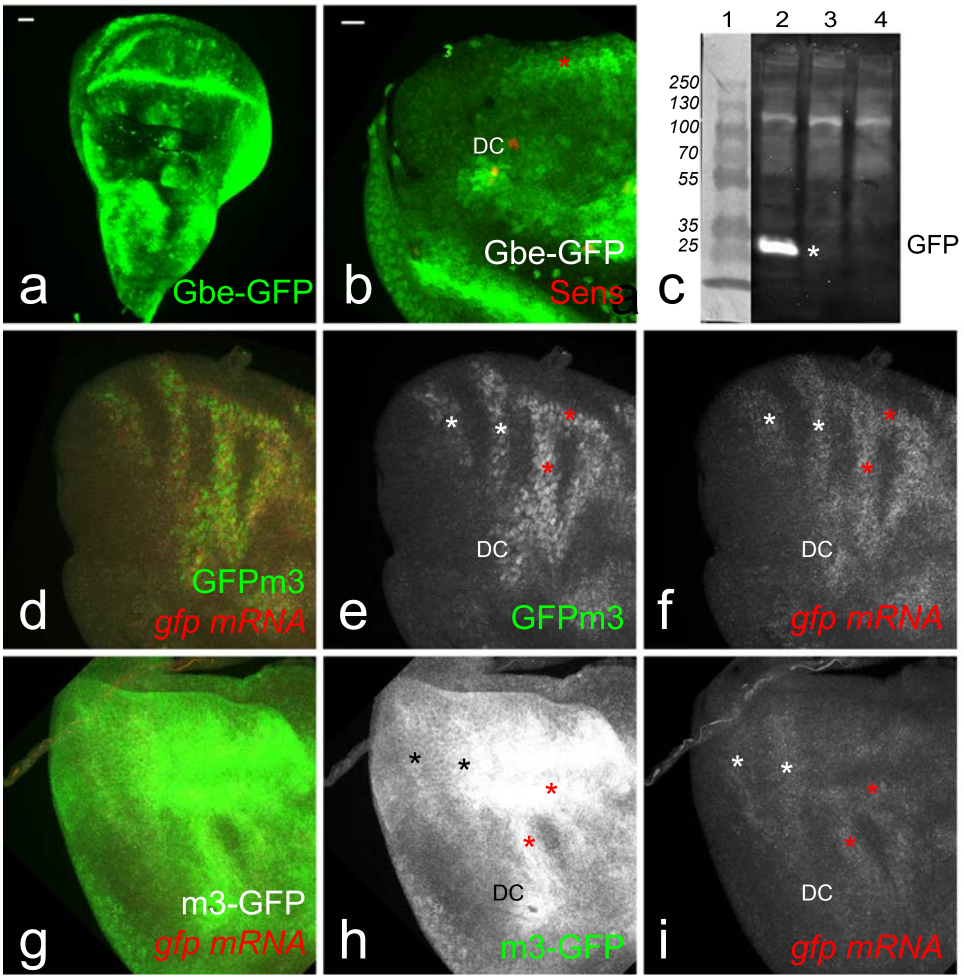

Supplementary Figure 2: GFPm3 appears to be less stable than GFP

a,b) Analysis of the activity pattern of a synthetic reporter [1] (Gbe-GFP, green) in third instar imaginal wing disc (a) and in the pupal notum at 2h apf (b). In wing discs (a), the GFP pattern differed from those seen for the E(spl)-HLH reporters considered individually. This can be explained by the context-specific response of the *E(spl)-HLH* genes to Notch. In the pupal notum (b), the expression of Gbe-GFP is hardly detected in the notum region where stripe patterning takes place (DC, position of the DC macrochaetes; Sens, red). This suggests that this synthetic Notch reporter may not always properly detect dynamic Notch signaling.

c) Western blot analysis showing that GFP from brain complex extracts of m3-GFP larvae (transcriptional reporter; lane 2) appeared to accumulate at a much higher level than GFPm3 (protein fusion; lane 3): while a strong GFP band was detected, GFPm3 remained

undetectable in these conditions. A negative control (wild-type larvae) is shown in lane 4 (Molecular weight markers, lane 1).

d-i) GFP (green in d,e; m3-GFP transcriptional reporter) accumulated at a higher level than GFPm3 (green in d,e; fusion protein) in 2h apf nota (compare d and h; DC indicates the position of the DC cluster). By contrast, no clear difference was observed at the mRNA levels (gfp FISH probe, red; compare f and i). Since GFPm3 (fusion protein) and GFP (replacing m3) are expressed under the control of the same *m3* regulatory elements, we suggest that GFPm3 is less stable than GFP. This is consistent with the notion that E(spl)-HLH proteins are generally unstable. See Figure 4 for a complete description of the expression pattern of the *E(spl)m3-HLH* gene in 2h apf nota.

Scale bars, 15μm (a) and 10 μm (b: b, d-i). Representative images of >6 discs from >2 experiments are shown. A representative blot from 2 experiments is shown.

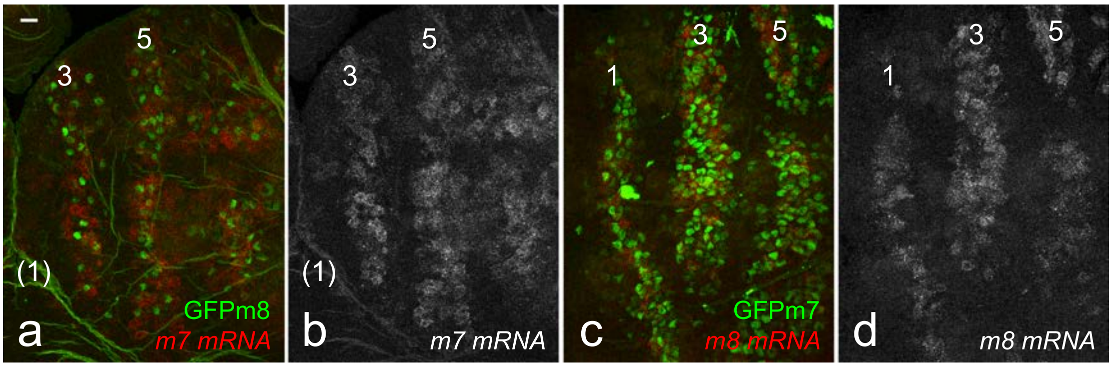

Supplementary Figure 3: m7 and m8 are co-expressed in the pupal notum

The patterns of GFPm8 (a; anti-GFP, green) and GFPm7 expression (c; anti-GFP, green) were compared with the distribution profiles of the *m7* (a,b; red in a) and *m8* (c,d; red in c) mRNAs in pupal nota at 8h apf. This analysis showed that these two *E(spl)-HLH* genes are co-expressed. Numbering refers to proneural stripes. Note that stripe 1 is not visible in panels a,b due to tissue folding at the midline; its position is indicated by (1).

Scale bar (a), 10µm. Representative images of >6 samples from >2 experiments are shown.

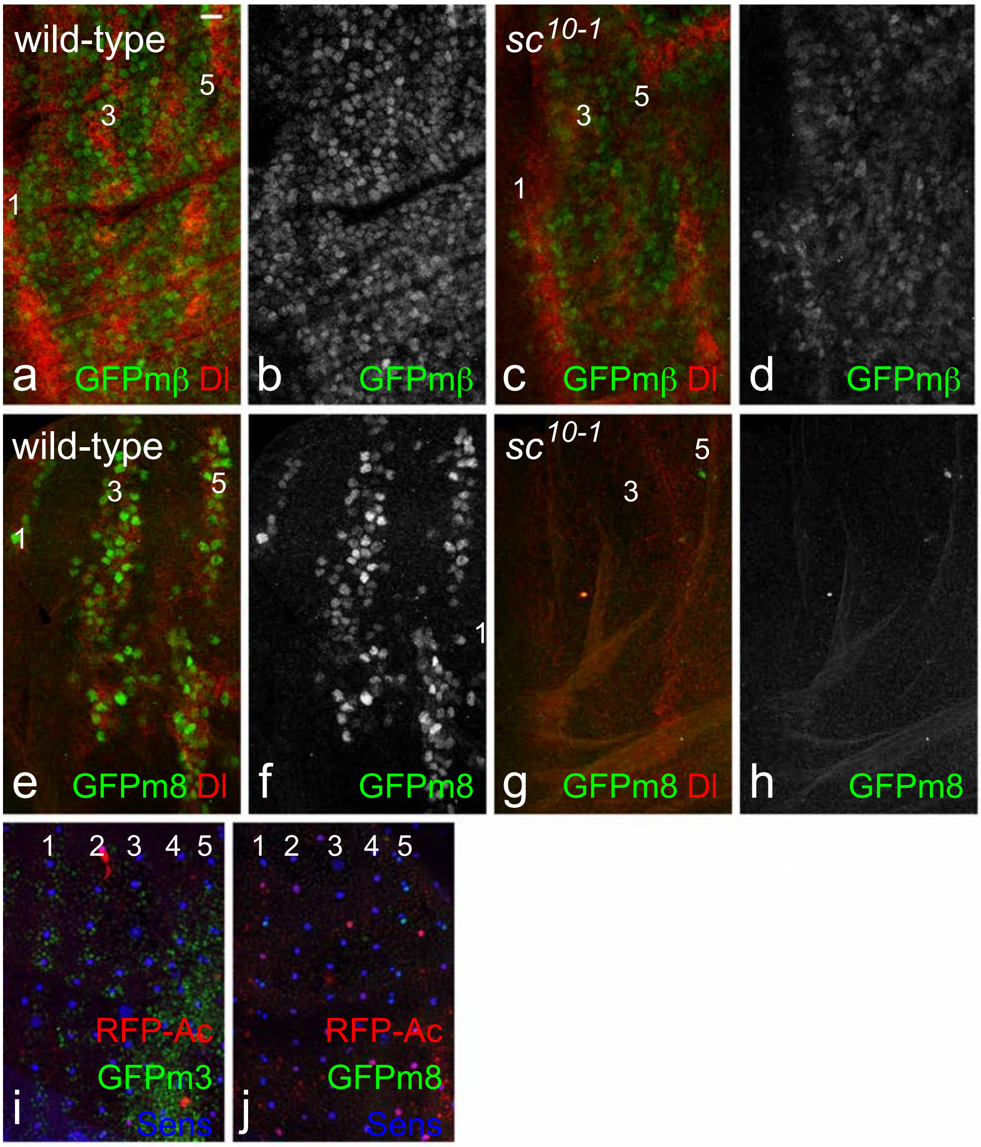

Supplementary Figure 4: m8, but mβ, is expressed in a *ac/sc*-dependent manner

a-h) Pattern of expression of the GFPmβ (a-d) and GFPm8 (e-h) reporters (GFP, green; Dl, red) in the notum of wild-type (a,b,e,f) and sc^{10-1} (c,d,g,h) 8h apf pupae. The expression of GFPmβ was largely independent of Ac and Sc (a-d). In contrast, GFPm8 was not expressed in the absence of Ac and Sc, (e-h).

i,j) At 14h apf, when proneural activity has returned to low levels (RFP-Ac, red), GFPm3 (i; GFP, green) was still expressed in rosettes of cells surrounding SOPs (Sens, blue) whereas GFPm8 (j) was no longer detected.

Scale bar (a), 10µm. Representative images of >6 samples from >2 experiments are shown.

Supplementary Table

allele	gRNAs
m3[CR1]	GTTGCGCGTTCCACCGATTG<br>GTGGGCGGTACTGGTTGAGG
mβ[CR1]	CGTTCCACTAGATTCGGACT<br>GTGGTTCCCTCGCTGCCCAT
m3[GFP-KI]	GCGTGTGTGAGTGATCATGC<br>GTAAACAACATTGGCGGACT
m7[FRT] *V5-m8*[FRT-KI]	AGCACGAGACGCTCTCGCGG<br>GGGACCAATCCCTTAGTTGG

Supplementary Table 1: gRNAs used for CRISPR-based engineering

Supplementary Methods

GFP-E(spl) reporters

These were generated by recombineering in a two- step process. In a first step, we inserted a dual kanamycin and streptomycin selection marker, constructed based on the kanamycin resistance gene *neo* and the bacterial *rpsL*$^{+}$ gene, 5' to the start codon of the different E(spl)-HLH protein, using the following primers:

rpslmbeta-f:

ccccccaataaaaaaaaaacaaaaccaaactacaacaaaggcctggtgatgatggcgggatcgttgtatatttcttg

rpslmbeta-r: ggtcttggacatctccatttccagaaccattcagaagaactcgtcaagaa

rpslmdelta-f: acaaacaatcaccacaccaaatcaaaacccattatacacaggcctggtgatgatggcggg

rpslmdelta-r: gctgagtcttggtcataaatctctgaccctgaacggccattcagaagaactcgtcaagaagg

rpslmgamma-f: ctacaaaaagaacaataagaaacacacaaaggcctggtgatgatggcggg

rpslmgamma-r: ggacatctcggacatttgtagcgacgacattcagaagaactcgtcaagaa

rpslm3-f: caatccttaaaatacacaacataaaacaacccaatcgatcggcctggtgatgatggcggg

rpslm3-r: taatattagggataagatccatvattgggcacgcccaattatcagaagaactcgtcaagaa

rpslm5-f: catttctacaaatcttccaaaacaaaaaacacattacaaaggcctggtgatgatggcggg

rpslm5-r: tggagacgaatgtggtgctgttgttgctctgtggtgccattcagaagaactcgtcaagaagg

rpslm7-f: catttacctaaacgcaacaaataaacaaaacaaacacacaggcctggtgatgatggcggg

rpslm7-r: ggtactgataggttttcgacatctcgtatttggtggccattcagaagaactcgtcaagaagg

rpslm8-f: caaaaatttttaaaactacgtaacctactaccaaaacaaaaggcctggtgatgatggcggg

rpslm8-r: cttcaccttctggtagatctgggtcttggtggtgtattccattcagaagaactcgtcaagaagg

In a second step, the dual kanamycin and streptomycin selection marker was replaced by the ORF of the sfGFP followed by a c-terminal GVG linker using the following primers:

gfpmbeta-f: cccccaataaaaaaaaaacaaaaccaaactacaacaaaatggtgagcaagggcgaggagctgttcaccgggg

gfpmbeta-r: ggtcttggacatctccatttccagaaccatgccaacacccttgtacagct

gfpmdelta-f: acaaacaatcaccacaccaaatcaaaacccattatacacaatggtgagcaagggcgaggagc

gfpmdelta-r: gctgagtcttggtcataaatctctgaccctgaacggccatgccaacacccttgtacagctcg

gfpmgamma-f: ctacaaaaagaacaataagaaacacacaaaatggtgagcaagggcgagga

gfpmgamma-r: ggacatctcggacatttgtagcgacgacatgccaacacccttgtacagct

gfpm3-f: caatccttaaaatacacaacataaaacaacccaatcgatcatggtgagcaagggcgaggagc

gfpm3-r: taatattagggataagatccatattgggcacgcccaattaggatcccttgtacagctcat

gfpm5-f: catttctacaaatcttccaaaacaaaaaacacattacaaaatggtgagcaagggcgaggagc

gfpm5-r: tggagacgaatgtggtgctgttgttgctctgtggtgccatgccaacacccttgtacagctcg

gfpm7-f: catttacctaaacgcaacaaataaacaaaacaaacacacaatggtgagcaagggcgaggagc

gfpm7-r: ggtactgataggttttcgacatctcgtatttggtggccatgccaacacccttgtacagctcg

gfpm8-f: caaaaatttttaaaactacgtaacctactaccaaaacaaaaatggtgagcaagggcgaggagc

gfpm8-r: cttcaccttctggtagatctgggtcttggtggtgtattccatgccaacacccttgtacagctcg

A similar strategy was used for the m3-GFP transcriptional reporter, except that the sequence of the ORF of the *m3* gene was replaced by the dual kanamycin and streptomycin selection marker in step 1. The following primers were used:

step1:

rpslm3-f: caatccttaaaatacacaacataaaacaacccaatcgatcggcctggtgatgatggcggg

rpslsfgfp-r:taatattagggataagatccatattgggcacgcccaattatcagaagaactcgtcaagaa

step2:

m3sfgfp-f: caatccttaaaatacacaacataaaacaacccaatcgatcatggtgtccaagggcgagga

m3sfgfp-r: taattgggcgtgcccaatatggatcttatccctaatattacttgtacagctcatccatgc

Donor plasmids for CRISPR-mediated homologous recombination

GFPm3 - The donor plasmid for GFPm3 was produced by Gibson assembly using a pBluescript SK+ digested by SpeI and NotI combined with gel-purified PCR products, which were obtained using the following primers and templates:

template: pACMAN GFPm3 [2]

• amplification of the left homology arm of the *m3* gene:

LHarm-f: gcttgatatcgaattcctgcagcccgggggatccactagtccatcggatcgcatcacaatcc

LHarm-r: ctcccggccacaaagcttttgcatgatcactcacacacgctcccaggcactcgggcccaag

• amplification of GFP:

gfpm3-f: cttgggcccgagtgcctgggagcgtgtgtgagtgatcatgcaaaagctttgtggccgggagagc

gfpm3-r:

agatctgggggcggggccggggttctcctccacgtcgccggcctgcttcagcagggagaagttggtggcgccggaccagggcctcc
agacgggc

• amplification of the right homology arm of the *m3* gene:

RHarm-f: tgtatgctatacgaagttattgccccaataaattacctttaattgaac

Rharm-r: aagggaacaaaagctggagctccaccgcggtggcggccgctggaatccatggaaaacagccg

template: pCRII-GFP3Sdt [3]

• amplification of the loxP-3xP3-RFP-loxP fragment)

rfp-f:
gaagttcctattctctagaaagtataggaacttctttagtccgccaatgttgtttacataacttcgtataatgtatgctatacgaagttat
gtcgacgaattcgcgg

rfp-r: aaaggtaatttattggggcaataacttcgtatagcatacattatacgaagttatactagagagcttcgca

FRT-m7-V5m8-FRT - The donor plasmid for the FRT-m7-V5m8-FRT allele was produced by recombineering of the attB-P[acman]-Ap BAC covering the *E(spl)-C* in a four- step process.

step 1: the PAM sequence targeted by gRNA5' was mutated and a first FRT sequence was inserted using the following primers:

FRT1m7rpsl-f:
gctccgcaggtggtggttcttcggaggctccgcagctctctcttctctctttctccgagcagcgtgcgctgagcacgagaggcctggtg
atgatggcggg

FRT1m7rpsl-r:
agatgttgactgcacgacgattgcgttcgaaatgttgcattcgtctccggttaccgctgcttttatagctgcacgtgccgtcagaagaa
ctcgtcaagaa

FRT1m7-f:
tctttctccgagcagcgtgcgctgagcacgagagaagttcctattctctagaaagtataggaacttccggcacgtgcagctataaaag
cagcggtaaccg

step 2: a V5 tag was introduced at the C-terminus of E(spl)m8-HLH using the following primers:

m8V5rpsl-f:

ccgtcgagcaggcagcaacaagccgaatacaaaatttttcaaaaatttttaaaactacgtaacctactaccaaaacaaaaggcctg gtgatgatggcggg

m8V5neo-r:

ttcatgcgggcacgtcgctggcgctccagcattggcttcttcaccttctggtagatctgggtcttggtggtgtattccattcagaagaac tcgtcaagaa

V5m8-f:

taaaactacgtaacctactaccaaaacaaaaatggtcggaaagcccatccctaatcctttgctgggcctggacagcacgggtgttgg catggaatacaccaccaagacccagatctacca

<u>step 3:</u> the PAM sequence targeted by gRNA3' was mutated, a second FRT site was introduced along with the 3xP3-RFP flanked by loxP sites using the following primers:

FRT2m8rpsl-f:

aaacaaatataaaccaatttaaacaaatatcaaaacaatttactttattgggatgttgggaggggatctaagggaccaatggcctgg tgatgatggcggg

FRT2m8neo-r:

tttccattgtcagagcagagatatattatttgatatttcctaattggtaataatttaattaccaataaggattccaattgtcagaagaac tcgtcaagaa

FRT2m8-f:

ttattgggatgttgggaggggatctaagggaccaatgaagttcctattctctagaaagtataggaacttcataacttcgtataatgtat gctatacgaagttatgtcgacgaattcgcgg

FRT2m8-r:

cagagcagagatatattatttgatatttcctaattggtaataatttaattaccaataaggattccaattgataacttcgtatagcataca ttatacgaagttatactagagagcttcgca

<u>step 4:</u> the genomic fragment corresponding to the donor template was fetched from the recombineered BAC into the pCR2.1 plasmid using the following primers:

pCR2FRTm7m8-f:

cggctgatctgtgtgaaatcttaataaagggtccaattaccaatttgaaactcagtttgcggcgtggcctttggttaaaaaatgagctg a

pCR2FRTm7m8-r:

ctcgctcctgctcttgtcaattatgaatgctgaattgtagcttctgttgaacacttgggccctgggtttccttccgcttcctcgctcact

Supplementary references

1. Furriols, M. & Bray, S. A model Notch response element detects Suppressor of Hairless-dependent molecular switch. *Curr Biol* **11**, 60-64 (2001).
2. Corson, F., Couturier, L., Rouault, H., Mazouni, K. & Schweisguth, F. Self-organized Notch dynamics generate stereotyped sensory organ patterns in Drosophila. *Science* **356** (2017).
3. Perez-Mockus, G., Roca, V., Mazouni, K. & Schweisguth, F. Neuralized regulates Crumbs endocytosis and epithelium morphogenesis via specific Stardust isoforms. *J Cell Biol* **216**, 1405-1420 (2017).
